# Urinary excretion of platinum, arsenic and selenium of cancer patients from the Antofagasta region in Chile treated with platinum-based drugs

**DOI:** 10.1186/1756-0500-5-207

**Published:** 2012-04-30

**Authors:** Domingo A Román, Isabel Pizarro, Lidia Rivera, Carolina Torres, Juan Ávila, Pedro Cortés, Marjorie Gill

**Affiliations:** 1 Bioinorganic and Environmental Analytical Chemistry Laboratory, Chemistry Department, Faculty of Basic Science, University of Antofagasta, Antofagasta, Chile; 2 Oncological Center, Antofagasta Regional Hospital, Antofagasta, Chile

**Keywords:** Cancer, Platinum-based drugs chemotherapy, 24-hours urine samples, Heavy metal urine analyses, Platinum, Arsenic, Selenium, Inter-element relationships

## Abstract

**Background:**

Arsenic exposure increases the risk of non-cancerous and cancerous diseases. In the Antofagasta region in Chile, an established relationship exists between arsenic exposure and the risk of cancer of the bladder, lung and skin. Platinum-based drugs are first-line treatments, and many works recognise selenium as a cancer-fighting nutrient. We characterised the short-term urinary excretion amounts of arsenic, selenium and platinum in 24-h urine samples from patients with lung cancer and those with cancer other than lung treated with cisplatin or/and carboplatin. As - Se - Pt inter-element relationships were also investigated.

**Results:**

The amounts of platinum excreted in urine were not significantly different between patients with lung cancer and those with other cancers treated with cisplatin, despite the significant variation in platinum amounts supplied from platinum-based drugs. In general, the analytical amounts of excreted selenium were greater than those for arsenic, which could imply that platinum favours the excretion of selenium. For other types of cancers treated with drugs without platinum, excretion of selenium was also greater than that of arsenic, suggesting an antagonist selenium-anti-cancer drug relationship.

**Conclusions:**

Regards the baseline status of patients, the analytical amounts of excreted Se is greater than those for As, particularly, for cisplatin chemotherapy. This finding could imply that for over the As displacement Pt favours the excretion of Se. The analytical amounts of excreted Se were greater than those for As, either with and without Pt-containing drugs, suggesting an antagonist Se-anti-cancer drug relationship. However, it seemed that differences existed between As - Se - Pt inter-element associations in patients treated for lung cancer in comparison with those treated for cancer other than lung. Therefore, knowledge obtained in this work, can contribute to understanding the arsenic cancer mechanism and the As - Se - Pt inter-element association for lung cancer and other types of cancer, which in some cases respond at a linear mathematical model.

## Background

There has been deterioration in the environmental health of people living in the Region II (Antofagasta) in Chile. This condition seems to be associated with medical geology characteristics of this part of the Atacama Desert ecosystem, geological structures in certain places on Earth influence human health [1,2] and the considerable mining and metallurgical activities carried out in this geographical zone, that which exposes the inhabitants to an increasing risk of cancer, cardiovascular diseases, and neurological diseases. The underlying cause could be the multi-metallic synergy of heavy metals [3]. However, arsenic (As) appears to be one of the main environmental stressors that affect the quality-of-life (QoL) of the population [4-7]. The Antofagasta region in Chile is an Andean highland–coastal desert ecosystem in which large-scale mining activities have been conducted for a long time [8]. These activities have affected the ecosystems and the QoL of the people living there [9].

Cancerous and non-cancerous diseases associated with As contamination are known in several parts of the world [10]. However, this association reaches alarming levels in the Antofagasta region, as evidenced by the incidence of cardiovascular diseases [5] and cancer [4,7,11]. The mortality rates due to cancer in the Antofagasta region are higher than those in any other population exposed to As in the world [12]. Most therapeutic treatments are based upon platinum (Pt)-based drugs [13].

There is a large body of literature on the role of trace elements in the development of cancer [14], particularly on the toxic effects of As on ecosystems and humans [15]. A wide variety of diseases, disorders and dysfunctions are related specifically to the effects of heavy-metal stress, as well as the imbalance of essential elements if humans are exposed to several metals [3]. However, the precious heavy metal Pt is of considerable interest, given that Pt pollution has increased in recent years because of its emission from automotive catalysts, its use in prostheses, and in Pt-based anti-tumour drugs [16,17]. Administration of Pt-based drugs has an impact at the cellular level, affecting metabolism, homeostasis, and physiology in humans [18,19]. The main side effects of the application of Pt drugs are loss of hearing (ototoxicity), kidney dysfunction (nephrotoxicity) [20], and resistance to anti-cancer drugs [21]. However, the metastasis [22] represents the principal disadvantage of these treatments.

Despite of the advances in cancer treatments, the cytotoxicity of chemotherapies continues to be an important problem. Undesirable effects arising from inaccurate calculation of the Pt-based dose drugs when the body surface area (BSA) method is applied [23], which can influence the As, Se, and Pt inter-element interactions and therefore the metal–creatinine ratios values and their clinical interpretation. Nevertheless, failure in correctly applying the BSA method does not account for the marked inter-patient variation in drug handling. Therefore, drug effects such as toxicity or consequences due to unexpected under-dosing are unpredictable [24].

Most toxicology studies focus on a single agent, but this approach does not reflect real-world scenarios in which humans are exposed to multiple chemicals. The toxicity of an element or compound may be reinforced or weakened through its interaction with other substances. Such interactions are, in general, classified as being ‘adversely additive’, ‘synergistic (greater than additive)’ or ‘antagonistic’ (less than additive or even acting as antidotes to one another). Antagonistic behaviour between As-Se and Pt-Se pairs has been observed in humans [25-29]. Hence, the level of Se in urine [30,31] could be particularly important when assessing exposure to heavy metals. Se would strengthen the capacity of antioxidant-protecting components of cells (e.g., nucleic acids) from damage of reactive oxygen species [22,30] through antioxidant Se-based enzymes such as glutathione peroxidase [31] and thioredoxin reductase, which require Se(IV) for their expression [28,32]. Several hypotheses have been stated to account for experimental data, which indicates that Se compounds have anti-cancer properties. However, even though evidence has been accumulating in support of the ‘Se chemo protective hypothesis’, definitive proof is lacking [33-38]. Otherwise, the vulnerability of the antioxidant-protecting components of cells can also result in side effects, metastasis [34,35] and resistance to Pt-based drugs [21].

Evidence suggests that As inhibits the potential anti-cancer effects of Se, and also its interaction with essential trace elements and other heavy metals such as copper, zinc, cadmium, mercury, tin, lead, nickel, cobalt, antimony, silver, gold and molybdenum [36,39]. In a study on the urinary excretion of Se and As in pregnant women from northern Chile, Vahter [40] found a significant association between the urine concentrations of these elements. Drugs that contain Pt (e.g., cisplatin, carboplatin) have been shown to increase the biliary excretion and distribution of Se in tissues, but Pt has not been shown to significantly influence the urinary or respiratory excretion of Se [41]. The distribution of Se in tissues is altered by cisplatin, which results in high concentrations of Se in the liver, kidney and plasma, and low levels of Se in the testicles and brain. The underlying mechanism of Se homeostasis is incompletely understood [20].

Studies looking at urinary excretion of Pt among patients treated with cisplatin have, on average, been conducted 6 months to 16.8 years after treatment [42]. With the purpose of obtaining knowledge on the behavior and the inter-relationships among the endogen arsenic-selenium pair and platinum supplied as cisplatin and/or carboplatin, we investigated the excretion of As, Se and Pt in 24-h urine samples after the infusion of Pt based-drugs in five cancer patient groups coming from the Region II (Antofagasta) - Chile. Short-term monitoring of urine after the first cycles of treatment with Pt-based drugs could be a good approach for studying the inter-element interaction among As, Se and Pt in patients whose cancer was probably triggered by As exposure. Such knowledge could contribute to: better understanding the cancer mechanism of As and mechanisms of Pt-based drugs based side effects [21] and resistance to these drugs [43]; improving the optimal doses calculation of the anticancer Pt-based drugs [23] and understanding better the resistance (or permissiveness) to metastasis [44].

## Methods

The work was carried out in accordance with the Health Service of Antofagasta (Antofagasta, Chile) according to ORD 4391 (20 August 2008); the study protocol was also authorised by the Ethics and Biosafety Committee of Antofagasta University (CEICREV/2008).

### Study population and collection of samples

Ten 24-h urine samples from cancer patients of the Antofagasta region were collected before Pt-based drugs chemotherapy was initiated. Ninety 24-h urine samples from cancer patients were collected immediately after the infusion of the Pt-base drugs; and ten 24-h urine samples were collected from patients subjected to chemotherapy drugs involving drugs that did not contain a metal. All samples were provided by the Oncological Centre of Antofagasta Regional Hospital (Region II, Chile). The 90 cancer patients they are distributed in the following cases: 32 lung cancer, 16 ovarian cancer, 12 cervical–uterine cancer, eight testicular cancer, five thymus cancer, three oesophageal cancer, three gastric cancer, three Hodgkin lymphomas, two vagina cancer, two bladder cancer, and one each of gallbladder, tonsil, salivary gland, and osteosarcoma cancers. The dose calculation for chemotherapy had been made by the BSA method [24]. Three of the four patients with ovarian cancer initially treated with cisplatin were again treated with cisplatin after 28 days, whereas the fourth patient was treated with carboplatin. Cancer others than lung was categorised as “others cancers”; only patients in this group were treated with carboplatin. Samples were collected between May and August 2005.

### Chemicals, solutions, materials and instruments

The chemicals employed were Instra (J.T. Baker, Phillipsburg, NJ, USA) and Suprapur (Merck, Whitehouse Station, NJ, USA), both of which were of trace-element grade. Solutions were prepared in deionised water that was subsequently distilled in quartz (conductivity, <0.5 μMHO/cm at 25°C). The deionisation was undertaken in a deioniser with two ion-exchange cartridges—a Metex 01506-45 and a Research 01506–35 (Cole-Parmer, Waltham, MA, USA)—connected in tandem.

The glass materials used were sequentially treated for 24 h with 2-M solutions of hydrochloric acid and nitric acid prepared from Merck pro analysis quality grade chemicals. After being washed with water, the glass materials were treated for another 24 h with a 0.025 M disodium salt of ethylenediamine tetraacetic acid (EDTA) solution prepared from Titriplex III (Merck), after which they were rinsed with water.

Pt(II) standard dilutions were prepared from a primary solution of Certipur (Merck) and those of Se(IV) were prepared from a primary solution of Titrisol (Merck); the concentration of both primary solutions was 1000 mgL^–1^. An intermediate 50 mgL^–1^ standard solution was made, which was used to prepare the working dilutions. Standard As solutions were prepared from a 1000 mgL^–1^ primary solution of disodium arsenate Titrisol (Merck), from which a 500 mgL^–1^ intermediate standard dissolution was prepared for the working dilutions. To generate arsenic hydride, 10 M chlorhydric acid solution was used, which was prepared from Instra (J.T. Baker). A 3% *p/v* sodium borohydride solution was prepared in a 1% *p/v* sodium hydroxide solution, both of which were Merck pro analysis grade quality chemicals.

Urine samples were transported in a cooler at 4–5 °C to an acclimatised and pressurised Pre-treatment Sample Room equipped with a laminar-flow hood (Labconco Purifier, class II; Labconco, Kansas City, MO, USA). Samples were microfiltered in a Nalgene system through a Millipore cellulose ester membrane (pore size, 0.45 μm; diameter, 47 mm; Millipore, Bedford, MA, USA). Samples were then stored at –20 °C. Immediately before analyses of metals were carried out, the samples were thawed and again microfiltered, this time through a Millipore cellulose ester membrane of pore size 0.22 μm protected by a borosilicate glass pre-filter (Advantec-MFS, Japan) (pore size, 0.7 μm).

Matrix-matched ionic strength surrogate urine was prepared by diluting subtidal seawater thrice with deionised glass-distilled water. As a reference, the measured average salinity of urine samples was 13 parts per thousand (ppt). This was measured in a previously calibrated Orion Ion Meter 1260 (Thermo Scientific, Waltham, MA, USA).

### Digestion of urine samples

Urine samples and controls were mineralised using the wet method in Teflon bombs with 10 ml of nitric acid (Instra; J.T. Baker, USA) for 2 h at 150 °C in a homemade ceramic oven with an internal temperature sensor and external temperature control. After the samples were cooled, the oven was opened and an additional 0.25 mL of perchloric acid and 0.50 mL of sulphuric acid were added to the Teflon bombs with the digested samples; both acids were of Suprapur grade (Merck). Samples were then reheated under the same conditions of time and temperature as described above. Finally, samples were transferred to Erlenmeyer flasks and heated at 300 °C under a gas extraction hood to eliminate excess acids. Sample volumes were made up to 50 mL with deionised water, and then micro-filtered through a 0.22-μm Millipore membrane with a 0.7-μm fibreglass pre-filter (Advantec-MFS, Japan).

To determine total As concentrations, 5-mL aliquots of samples and controls were digested to semi-reflux under a temperature programme between 85 °C and 300 °C [45]. After cooling, samples and controls were diluted volumetrically with 0.5 M HCl prepared from Instra quality grade chemical (J.T. Baker, USA).

### Determination of total Pt and Se in urine through inductively coupled plasma optical emission spectrometry (ICP–OES)

With the aim of meeting the matrix-matching conditions, aliquots of three samples of digested urine were titrated with NaOH secondary standard solution, yielding an average acidity equivalent to a pH of 2. To make compatible the matrix-matching conditions of the analytical blanks, seawater was diluted 8-fold, 10 mL of sample was diluted to a final volume of 50 mL and, when necessary, the solution was adjusted to pH 2 with Instra nitric acid (J.T. Baker, USA). The bimetallic Pt–Se standard was also subsequently prepared in this medium.

ICP–OES calibration curves were constructed from a 50 μg/mL Pt–Se bimetallic standard at the following Pt/Se concentrations: 100/100; 500/200; 1,000/300; 2,500/400; and 5,000/500 ng/mL. The bimetallic standard with the highest concentration was used to optimise the instrumental parameters. ICP–OES measurements were done on a GBC Integra XL Spectrometer (Integra, Canberra, Australia) equipped with a spray trace nebuliser chamber and a 200 μL Micromist nebuliser (Glass Expansion, Melbourne, Australia). Analytical-grade 5 N_2_ and Ar gases were used. The optimised experimental conditions are shown in Table 1. The baseline was adjusted with the analytical blank solution, and quality-control (QC) tests were made by evaluating the recovery of Pt–Se internal standard additions over blank and real samples. Before their measurement, urine samples of patients underwent the procedures and analytical methods described above.

**Table 1:** Instrumental optimised conditions for urine analysis of As, Se and Pt by means HGAAS and ICP–OES

**As HG–AAS**	
Bandpass width	2 nm
Bandpass height	Normal
Lamp current	8.0 mA
Backup current	30 mA
Temperature EHG 3000	920°C
N_2_ flow	45–50 mL/min
Time signal stabilisation	60 s
Reading time	10 s
**Pt–Se ICP–OES**	
Spectral line of Pt	265.945 nm
Spectral line of Se	196.026 nm
Nebulising flow	0.40 L/min
Line detection height	6.8 mm
Pump	17.0 rpm
Power	1200 W
Auxiliary gas	0.6 L/min
Ar feed	12.0 L/min
Nebulising chamber	Spray trace; Micromist nebuliser at 200 μL/min

### Determination of total As in urine by hydride-generation atomic absorption spectrometry (HGAAS)

Determination of As was undertaken using an atomic absorption spectrophotometer (GBC 909 AA; GBC Victoria, Australia) equipped with a continuous-flow hydride generator (GBC HG 3000) and a electrothermal atomisation system (GBC EHG 3000). GBC quartz cells and an As-boosted current lamp from Photron (Narre Warren, Victoria, Australia) were also used. The optimized conditions for measuring As through HGAAS are presented in Table 1. Multiple-standard-addition methodology was applied, for which an intermediate 500 mg/L standard solution was prepared in 0.5 M HCl from a primary standard solution (Titrisol; Merck). Additions from this solution were made to surrogate-sample urine, which had been made up to 2.0 μg/L in As. The addition volumes were 50, 100, 150 and 200 μL; the concentrations added were therefore 2, 4, 6 and 8 ng/mL, respectively.

The baseline was adjusted with the analytical blank solution i.e., diluted surrogate urine. QC tests were conducted to evaluate the recovery of As from As-spiked real samples and measurements of secondary standards. Urine samples of patients underwent the procedures and analytical methods described above.

### Urine and serum creatinine levels and calculation of creatinine clearance

Levels of urine and serum creatinine were measured using the Jaffé method [46]. Creatinine clearance rate (CrCl) was calculated using the Cockcroft–Gault equation. Serum creatinine is the blood clearance or endogenous blood creatinine depuration [47], their values were provided by the Oncological Center of Antofagasta Regional Hospital. Urine and serum CrCl values are used to evaluate renal dysfunction. The estimated CrCl for normal renal function should be >80 mL/min [48].

### Statistical analyses

Statistical analyses were conducted using the Statistica 9.1 computer programme (StatSoft, Tulsa, OK, USA). *p* < 0.05 was considered significant.

## Results and discussion

### Determination of Pt, Se and As

The calibration curves for ICP–OES determinations of total Pt and Se concentrations in urine were linear. In the case of Pt, the parameters of the calibration equation were: intercept = 93.45 counts · s^–1^; slope of 1.079 counts × s^–1^/concentration; and r = 0.9998. For Se, the parameters were: intercept = 55.47 counts · s^–1^; slope = 1.720 counts × s^–1^/concentration; and r = 0.9880.

The resulting parameters for the standard-addition methodology applied to As urinary analyses were also analytically suitable: intercept = 0.0375 uA; slope = 0.01615 uA/concentration; and r = 0.9977. The results of the QC test and analytical validation are presented in Table 2.

**Table 2:** Traceability and quality control of urinary analyses of As, Se and Pt in cancer patients

**Analyte**	**N**	**Added (ng/mL)**	**Found ng/mL)**	**Yield (%)**	**± RSD (%)**	**RE (%)**	**DL (ng/mL)**
Pt	11	1000	971.0	97.1	5.7	2.9	7.7
Se	11	300	290.9	97.0	6.3	3.0	5.4
As	11	4	3.87	96.7	6.1	3.3	0.22

RSD relative standard deviation; RE relative error; DL detection limit.

The analytical validation and QC results for Pt and Se by ICP–OES and of As by HGAAS analyses in 24-h urine samples of cancer patients indicated that the techniques and methodologies applied in the work were analytically suitable. Surrogate urine prepared from contaminant-free seawater was important for optimisation of the analytical parameters and appropriate representativeness of the results.

### Inter-element relationship of Pt, As and Se in urine of cancer patients from the Antofagasta region of Chile treated with Pt-based drugs

Recent developments in multi-elemental analytical techniques and methodologies have enabled exploration of inter-elemental relationships and toxicities of essential elements and heavy metals, that which coexist in environmental and biological matrices [49-54]. Knowledge in this area is mainly based upon single elements; a better-known case is that of As, which has been associated with several diseases, including cancer [55-58]. However, it has become increasingly clear that simple, single-element models are often inadequate to explain the relationship between disease and nutrition, element toxicity, or deficiency of elements in food. Improved understanding of the synergistic and/or antagonistic interactions between trace elements is needed [59]. A multi-elemental hypothesis may be a better approach to understand these relationships.

Renal function before and after cancer treatment with Pt-based drugs is an important parameter. Before starting anti-cancer treatment, it is advisable that the CrCl is >60 mL/min [47,60]. Patients with lower values have an increased risk of severe suppression of the bone marrow. The CrCl for normal renal function is > 80 mL/min [47,61]. The CrCl values before Pt-based drug chemotherapy for the control group 2 (baseline patients; see below) were 93.3–114.6 mL / min; serum and urine creatinine values were 0.6–0.9 and 0.8–1.1 mg / dL, respectively. Evidently, Pt-based drug chemotherapy impacts the CrCl and the metal/creatinine quotient values, both parameters are considered controversial [62,63]. According this approach, after chemotherapy, 82 of 90 cancer patients had kidney damage.

### Statistical parametric treatment

Tables 3 and 4 shows the main parametric statistic results found in 24-h urine samples of five cancer patient groups under study from Region II (Antofagasta). That is: 1) lung cancer treated with cisplatin; 2) cancer other than lung cancer treated with cisplatin; 3) cancer other than lung cancer treated with carboplatin; 4) cancer other than lung treated with drugs not containing metals (control 1); and 5) lung and other than lung cancer before the chemotherapy began, which can be considered the baseline group (control 2). The number of patients in group 3 and in each one of the control groups was fewer to 11 cancer cases, respectively. Normal distribution of the data was proven by applying the Shapiro-Wilk normality test. For groups 1–2 with more than 11 cancer patient cases, the Shapiro–Wilk normality test was also applied. One-way ANOVA (*p* ≤ 0.05) test were applied to determine the level of significance of the data. If the data of groups 1, 2 followed a normal or a non normal distribution, the most representative group value considered was the mean or the median, respectively. In Tables 3 and 4 the number of As and Se results are less than the total number of cancer types (n), because in some urine samples the amount of elements were below the detection limit of the applied analytical technique.

**Table 3:** Supplied amounts of Pt and Pt, As and Se in 24-h urine results for the group patients treated with Pt-based drugs and control group treated without Pt-based drug and the baseline group (a)

**Groups**	**Type of cancer**	**Parameter**	**Pt**_**Supp**_**(mg)**	**Pt (μg)**	**Se (μg)**	**As (ng)**
Cisplatin (Group 1)	Lung	X	163.0	35.3	3.62	127.0
		Med	156.0	28.2	1.28	23.5
		DS	66.6	25.9	6.45	267.0
		n	32	32	24	21
		Min	39.1	3.94	0.208	1.88
		Max	263.0	90.4	22.0	1180.0
Cisplatin (Group 2)	Other	X	174.0	40.1	1.35	216.0
		Med	120.0	32.5	1.22	71.0
		DS	324.0	36.1	0.717	424.0
		n	47	47	36	31
		Min	31.6	0.760	0.354	18.8
		Max	2297.0	175.0	2.84	2300.0
Carboplatin (Group 3)	Other	X	695.0	262.0	1.59	40.8
		Med	a	a	a	a
		DS	189.0	201.0	0.711	46.9
		n	11	11	9	6
		Min	433.0	9.80	0.516	2.53
		Max	917.0	680.0	2.52	121.0
Control 1 (b) (Group 4)	Other	X			1.77	62.0
		Med			a	a
		DS			1.14	59.4
		n			7	2
		Min			0.333	20.0
		Max			3.29	104.0
Control 2 (c) (Group 5)	Lung and other	X			1.47	48.2
		Med			a	a
		DS			0.58	1.56
		n			9	2
		Min			0.45	47.1
		Max			2.16	49.3

Amounts of supplied Pt and Pt, As and Se in 24-h urine samples.

Supp, Supplied; X, mean; Med, Median; SD, Standard deviation; n, number of cases; Min, Minimum value; Max, Maximum value; (a) due the small number of cases, the median values were not reported; (b) non platinum drugs treatment; (c) patients without chemotherapy (two gastric cancer, two bladder cancer and five lung cancer).

**Table 4:** **Pt, As, and Se amounts in 24-h urine as metal · (g creatinine)**^**-1**^**results for the group patients treated with Pt-based drugs and control group treated without Pt-based drug and the baseline group (a)**

**Groups**	**Type of cancer**	**Parameter**	**μg Pt/g Cr**	**μg Se/g Cr**	**ng As/g Cr**
Cisplatin (Group 1)	Lung	X	1.05	143.0	5.57
		Med	1.06	49.0	2.08
		DS	0.625	263.0	8.94
		n	32	24	21
		Min	0.136	7.0	0.360
		Max	2.52	1028.0	31.1
Cisplatin (Group 2)	Other	X	1.39	83.9	7.96
		Med	0.969	40.7	2.55
		DS	1.59	186.0	17.1
		n	47	36	31
		Min	0.110	11.3	0.280
		Max	7.26	1136.0	91.2
Carboplatin (Group 3)	Other	X	13.3	156.0	10.1
		Med	a	a	a
		DS	9.76	182.0	18.5
		n	11	9	6
		Min	0.780	25.5	0.40
		Max	32.5	484.0	47.7
Control 1 (b) (Group 4)	Other	X		51.6	
		Med		a	
		DS		33.7	
		n		7	
		Min		5.30	
		Max		92.3	
Control 2 (c) (Group 5)	Lung and other	X		61.9	
		Med		a	
		DS		20.4	
		n		9	
		Min		28.1	
		Max		90.4	

Pt, As and Se amounts in 24-h urine samples expressed as metal (g creatinine)^–1^

Supp, Supplied; X, mean; Med, Median; SD, Standard deviation; n, number of cases; Min, Minimum value; Max, Maximum value; (a) due the small number of cases, the median values were not reported; (b) non platinum drugs treatment; (c) patients without chemotherapy (two gastric cancer, two bladder cancer and five lung cancer).

Although the number of patient with cancer of the control groups was low, one can observe that in both cases the Se levels tend to be higher than those of As. However, in control group 2, the range of As and Se levels tends to be comparable or slightly smaller to than those of control group 1. In Region II (Antofagasta) Se concentrations have been shown to be higher than those As in the umbilical cords and placentas of normal and malformed newborn babies, and in tissues of patients’ subjected to cardiovascular surgeries [64,65]. This characteristics was also observed in the present study, inasmuch that the Se and As concentrations in group 3 were comparable with those of control 1 and control 2 groups.

**Figure 1:**
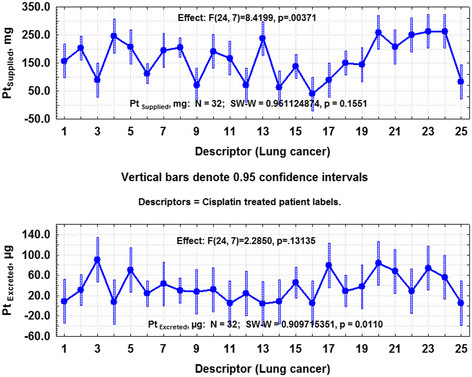
**a. One-way ANOVA plot (*p*≤ 0.05) for supplied platinum to lung-cancer patients treated with cisplatin. b.** One-way ANOVA plot (*p* ≤ 0.05) for excreted platinum from supplied platinum-based drug to lung-cancer patients.

Figure 1 shows the one-way ANOVA profiles (*p* ≤ 0.05) for calculated drug supplied Pt and Pt in 24-h urine samples from lung-cancer patients treated with cisplatin (group 1); the Shapiro–Wilk normality test parameter are also shown. The one-way ANOVA urine profiles for Se of patients other than lung cancer treated with cisplatin (group 2) did not exhibit significant variations (*p* ≤ 0.05), but urine profiles of As did (*p* ≤ 0.05). In both cases, the data did not follow a normal distribution.

Regarding the calculated drug-supplied platinum and the Pt, Se and As in urine, the results presented in Table 3 indicate that the decreasing tendency of supplied Pt was: carboplatin (cancer other than lung, 695 mg) > cisplatin (lung cancer, 163 mg) > cisplatin (cancer other than lung, 120 mg); the tendency of Pt amount in urine was the same as that of supplied Pt. The tendency of excreted Se amount in urine was carboplatin (cancer other than lung, 1.59 μg) > cisplatin (cancer other than lung, 1.22 μg) ≈ cisplatin (lung cancer, 1.28 μg). With respect to excreted As amounts in urine, the tendency was cisplatin (cancer other than lung, 71 ng) > carboplatin (cancer other than lung, 40.8 ng) > cisplatin (lung cancer, 23.5 ng). Hence, with Pt –base drugs and chemotherapy not involving metal-base drugs, Se amounts were always higher than As amounts in urine. This inter-element relationship suggests that, in general, anti-cancer drugs induce higher secretion of Se than of As, and that this relationship was independent of the nature of the drug used in the treatment.

The amounts of Pt, As and Se in 24-h urine samples from cancer patients expressed as metal/g creatinine showed the same distribution tendency and statistical significance as non-normalised values (Table 4). The comparison with metal/g creatinine values for healthy subjects (i.e., As, 33.3 μg/g creatinine [66,67]; Se, (women), 9.8 μg/g creatinine; Se (men), 13.5 μg/g creatinine [68]; and Pt, 3.3 ng/g creatinine [69]), along with the results obtained in this work, revealed that the values for cancer patients were lower than those for healthy persons. However, the values obtained for Se and Pt were higher than the values for healthy persons. The comparison of metal/g creatinine values between cancer patients from this work with those in other studies i.e., As, 116 μg/g creatinine (patients with arsenicism without cutaneous lesions); As, 121.2 μg/g creatinine (patients with arsenicism with skin cancer) [66]; Se, 206.7 μg/g creatinine [70]; and Pt 0.30–4.18 μg/g creatinine [42], indicated that, in this work, metal/g creatinine-reported values were lower than those of persons with skin cancer reported in the literature. For Se, the values reported in the literature were higher than those reported here and, for Pt from cisplatin, the values were within the range of those reported previously. However, in the case of Pt from carboplatin, the metal/g creatinine values obtained in this work were approximately threefold higher than the values in the literature [42]. Owing to a multiplicity of factors involved in creatinine excretion, the analytical reproducibility of the heavy metal/creatinine quotient value is not satisfactory; this parameter is also poorly representative in the clinical scenarios [63].

For 24-h urine samples, there were no significant differences between the excreted Pt amounts for lung-cancer patients and for patients with cancer other than lung cancer treated with cisplatin despite the significant differences in the amounts of supplied Pt. Analytically, the excretion of As was less than of Se, which could suggest that Pt favours Se excretion.

### Multivariate statistical treatment

The cluster technique was applied to the involved variables (i.e., calculated amounts of Pt supplied through Pt-based drugs and the urinary amounts of Pt, As and Se excreted in 24-h urine after the cancer patients chemotherapy with cisplatin and carboplatin). Cluster analysis is a multivariate statistical methodology applied to datasets if there is a lack of information about the classes comprising the data. The basic objective is to group the variables by similarities [71]. The technique is based on two aspects: the way the distance between the variables is measured (metric) and the groupings or clusters (linkage or amalgamation rule) [72]. The technique was applied to the most representative sets of values in accordance the Shapiro–Wilk normality test. Ward’s method was applied with Pearson’s 1-r approximation to measure the distance between variables and the groupings or clusters.

**Figure 2:**
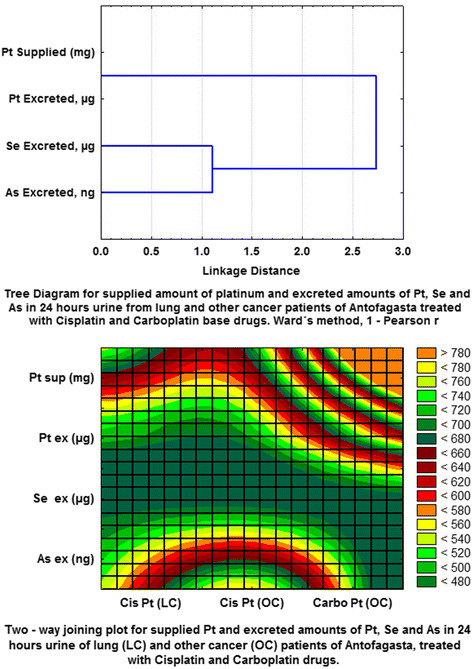
**a. Tree diagram of supplied platinum and amounts of excreted platinum, selenium and arsenic in 24-h urine samples of patients with lung cancer and cancer other than lung cancer treated with cisplatin- and carboplatin-based drugs. b.** Two-way joining plot for supplied platinum and excreted amounts of platinum, selenium and arsenic in 24-h urine samples for patients with lung cancer and cancer other than lung cancer treated with cisplatin and carboplatin.

Figure 2a shows the dendrogram of the variables related to 24-h urine values after the first cycle of treatment with cisplatin to lung-cancer cases as well as patients with cancer other than lung (groups 1, 2). In addition, 4 patients with ovarian cancer were considered after a second cycle of treatment. Carboplatin was used to treat only patients with cancer other than lung cancer, and only one patient had a second treatment cycle. Figure 2a shows two main groupings [Pt supplied - Pt excreted] and the amounts of As and Se excreted [Se, As excreted]. From application of the bidimensional grouping technique (Figure 2b), the quantitative extension of supplied Pt and the corresponding excretions of Pt (μg), Se (μg) and As (ng) in urine were compared. We concluded that urinary excretion of Pt after the infusion of cisplatin and carboplatin was low in the short term (even if more than one cycle drug was applied). Hence, Pt would initially be predominantly distribute and bio-accumulate in the tissues.

**Figure 3:**
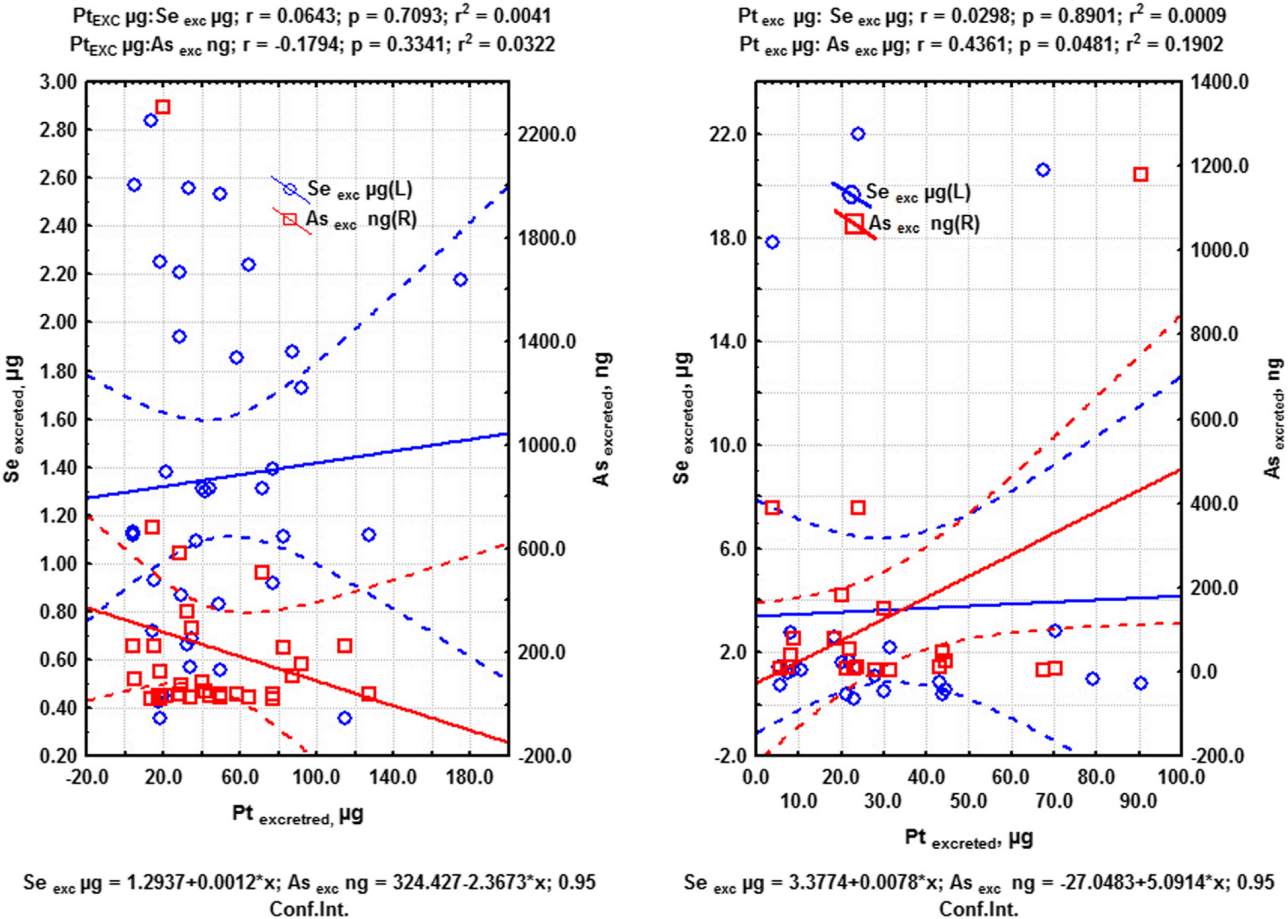
**a. Linear dispersion plots for excreted arsenic and selenium amounts versus excreted platinum amount for lung-cancer patients treated with cisplatin. b.** Linear dispersion plots for excreted arsenic and selenium amounts versus excreted platinum amounts for patients with cancer other than lung cancer treated with cisplatin.

Figures 3a (lung-cancer) and 3b (cancer other than lung cancer) show the statistical dispersion plots according to the linear correlation model for the excretion of Se and As compared with Pt excretion after cisplatin treatment (groups 1, 2). For lung-cancer patients (Figure 3a), the tendency of As excretion was contrary to the increased excretion of Pt. With regard to Se, only a small group of patients showed increased excretion of Se accompanied by increased excretion of Pt. For most cases, Se was distributed above and below the 95% confidence interval. According to our hypothesis, As excretion (cancer agent) was low compared with the Se excretion, and was negatively correlated with homeostatic excretion of Pt. Se excretion (protective agent against cancer) was comparatively higher than of As, but only a few cases were positively correlated with Pt excretion. Therefore, lung tumours would bind more strongly to As than Se.

For Pt from cisplatin infused in patients with cancer other than lung cancer (Figure 3b), excretion of As increased linearly with increased excretion of Pt, and the excretion of Se was similar to that described for lung-cancer patients treated with cisplatin (Figure 3a). According to our hypothesis, excretion of the cancer agent (As) compared with the excretion of Se was also low, but was positively correlated with the homeostatic excretion of Pt. In turn, the assumed protective agent against cancer (Se) did not correlate with Pt excretion, but its level of excretion was higher than seen in lung-cancer patients. In the case of patients with cancer other than lung cancer treated with carboplatin, the dispersion plot for excretion of As respect to excreted Pt exhibited inverse tendency (r = –0.2713), but Se did not correlate with excreted Pt.

Regard to supplied Pt through cisplatin to patients with cancer other than lung cancer, excretions of Se and As did not exhibit any tendencies. However, in the case of Pt supplied through cisplatin to lung-cancer patients, the excreted As tended to exhibit an inverse linear tendency (r = –0.2918) with respect to supplied Pt. In turn, with regard to supplied Pt by treatment with carboplatin in patients with cancer other than lung cancer, As tended to exhibit a direct linear tendency (r = 0.3987), and Se tended to exhibit an inverse linear tendency (r = –0.2388). These functional inter-element associations can be important for the hypothesis outlined in the presently work. Therefore, differences appeared to exist between the relationships and inter-element associations among As, Se and Pt for patients treated for lung cancer and those treated for cancer other than lung with Pt-based drugs. However, either with Pt-based drugs or those not containing Pt, the excretion of Se was greater than that of As. This phenomenon could enhance the effect of carcinogens such as arsenic, reducing the protective effect of Se, and thereby lead to metastasis [28,32,73].

Pt and As are not essential elements for humans, but Se is considered essential and an anti-cancer agent [32]. Consequently, the information obtained in this work about urinary levels of As, Se and Pt immediately after cancer patients were subjected to chemotherapy with Pt-based drugs, could contribute to a better understanding of the adverse effects of Pt, and help in the more accurate estimation of drug doses [24,74]. Consideration of urinary levels of carcinogens such as As and cancer-fighting nutrients such as Se could allow more individualised or dedicated strategies for predictive calculations of drug dosages to be applied to cancer patients.

A well-established correlation has been found between As exposure in the Antofagasta region of Chile and the risk of cancer of the bladder, lung and skin [75]. Consequently, urinary levels of As and Se after each treatment cycle with Pt-based chemotherapeutic drugs could be important for a better understanding of the carcinogenic mechanism of As exposure [76] and about the hypothetic cancer-protective effects of Se. Genetic and epigenetic processes appear to be involved in cancer mechanisms [77,78]. Epigenetic studies focus on the stress that As provokes in cells without affecting DNA, but directly influences the genome expression. In this complementary mechanism, As participates as an ‘opportunistic carcinogen’ that enhances the genotoxicity and mutagenicity of other environmental stressors [75]. During Pt-based drug treatments, patients are exposed to an acute impact of Pt. This subjects cellular homeostasis to deliberate stress by Pt, inducing toxic and, perhaps hormesic responses. Among cancer patients exposed chronically to arsenic and treats with Pt-drugs based, Se, As and Pt can participate synergistically in the regulated and unregulated activation of apoptosis, phagocytosis, and cellular homeostasis involved in toxicological and hormesis mechanisms [18,20,77,79-81].

## Conclusions

Overall, despite significant variation in Pt amounts supplied from Pt-based drugs, the amounts of Pt in 24 hours urine samples were not significantly different between patients with lung cancer and those with other cancers treated with cisplatin. With respect to the baseline status of patients, the analytical amounts of excreted Se were greater than those for As, particularly, for cisplatin chemotherapy. This finding could imply that, with regard to As displacement, Pt favours the excretion of Se. For other types of cancers treated with drugs without Pt, excretion of Se was also greater than that of As, suggesting an antagonist Se–anti-cancer drug relationship. Conversely, cisplatin displaces more As in patient with other cancers than lung cancer and more Se in patient with lung cancer. Whether or not they satisfied a linear mathematical model, there seem to have differences among the As - Se - Pt inter-element relationships according to cancer type.

The knowledge obtained in this work, can contribute to understanding the cancer mechanism of As and the As - Se - Pt inter-element association for lung cancer as well as other types of cancer. Elements with similar electron arrangements as the As - Se pair are often antagonists in biological systems [82], which can be rationalised by the Hard/Soft/Acid/Base principle, [83]. The antagonism and/or synergism among these elements can be intrinsic to the cancerous metalloid-dependent mechanism of the As-Se pair, and exogenic Pt participation in healing, cancer propagation or metastasis. Further research including more elements is in progress.

## Competing interests

The authors declare that they have no competing financial or non-financial interests.

## Authors’ contributions

DR conceived and designed the study, and carried out and interpreted the statistical treatment of the results. IP designed and undertook the sampling, directed the analytical work, and carried out instrumental interpretation. LR tested the analytical data and reviewed the adequacy of the analytical results. The graduate thesis of CT formed part of the present work. JA and PC participated in instrumental analytical measurements. MG obtained urine samples and participated in discussion of the work. DR and IP wrote the draft of the manuscript. All authors approved the final version of the manuscript.
